# Deoxycholate induces COX-2 expression via Erk1/2-, p38-MAPK and AP-1-dependent mechanisms in esophageal cancer cells

**DOI:** 10.1186/1471-2407-9-190

**Published:** 2009-06-17

**Authors:** Eileen Looby, Mohamed MM Abdel-Latif, Veronica Athié-Morales, Shane Duggan, Aideen Long, Dermot Kelleher

**Affiliations:** 1 Department of Clinical Medicine and Institute of Molecular Medicine, Trinity Centre for Health Sciences, Trinity College Dublin, St James's Hospital, Dublin 8, Ireland; 2 Department of Biochemistry and Immunology, Trinity College Dublin, Dublin 2, Ireland

## Abstract

**Background:**

The progression from Barrett's metaplasia to adenocarcinoma is associated with the acquirement of an apoptosis-resistant phenotype. The bile acid deoxycholate (DCA) has been proposed to play an important role in the development of esophageal adenocarcinoma, but the precise molecular mechanisms remain undefined. The aim of this study was to investigate DCA-stimulated COX-2 signaling pathways and their possible contribution to deregulated cell survival and apoptosis in esophageal adenocarcinoma cells.

**Methods:**

Following exposure of SKGT-4 cells to DCA, protein levels of COX-2, MAPK and PARP were examined by immunoblotting. AP-1 activity was assessed by mobility shift assay. DCA-induced toxicity was assessed by DNA fragmentation and MTT assay.

**Results:**

DCA induced persistent activation of the AP-1 transcription factor with Fra-1 and JunB identified as the predominant components of the DCA-induced AP-1 complex. DCA activated Fra-1 via the Erk1/2- and p38 MAPK while Erk1/2 is upstream of JunB. Moreover, DCA stimulation mediated inhibition of proliferation with concomitant low levels of caspase-3-dependent PARP cleavage and DNA fragmentation. Induction of the anti-apoptotic protein COX-2 by DCA, via MAPK/AP-1 pathway appeared to balance the DCA mediated activation of pro-apoptotic markers such as PARP cleavage and DNA fragmentation. Both of these markers were increased upon COX-2 suppression by aspirin pretreatment prior to DCA exposure.

**Conclusion:**

DCA regulates both apoptosis and COX-2-regulated cell survival in esophageal cells suggesting that the balance between these two opposing signals may determine the transformation potential of DCA as a component of the refluxate.

## Background

Bile acids are normal constituents of the gastro-intestinal tract where they act as trophic factors for the gut epithelium and as detergents for the absorption of cholesterol and fat-soluble vitamins [1,2]. Typical Western diets, rich in fat, are associated with increased incidence of gastro-intestinal cancer [3]. Dietary fat influences bile acid secretion as well as the composition of gut bacteria, which in turn determines the production levels of secondary bile acids [4-7].

While bile acids such as DCA cannot induce tumors, they are generally believed to be tumor promoters. The exact mechanism of their tumor promoting activity is uncertain but it is thought to involve alterations in cellular signaling cascades including activation of protein kinase C and gene expression systems [8]. Bile acids are known mediators of cellular stress [9] and have been proposed to induce apoptosis resulting in compensatory hyperproliferation, allowing for selection of apoptosis-resistant cells [10,11]. Bile acids are also known to induce survival mechanisms in parallel with apoptotic pathways in hepatocytes and colonic cells [12,13].

Over the past two decades there has been a significant increase in the incidence of Barrett's esophagus [14], a premalignant lesion leading to esophageal adenocarcinoma. This condition characterized by small intestinal metaplasia of esophageal epithelium is strongly associated with gastroesophageal reflux disease (GERD). Reflux of duodenal contents, of which bile acids are a major constituent, has been consistently associated with increased severity of both esophagitis and Barrett's esophagus [15,16]. Barrett's metaplasia has been reported in patients with bile reflux without any pathological acid reflux, as well as in patients on acid suppression therapy, highlighting the importance of refluxate components other than acid in esophageal cancer progression [17,18]. The concentration of bile acids, in particular unconjugated bile acids, in the refluxate of patients with GERD shows a strong direct correlation with the degree of esophageal mucosal damage [16,18]. Compelling evidence for the involvement of bile acids in Barrett's esophagus has also emerged from animal studies, where reflux leads to esophageal inflammation, increased mucosal thickening [19] and development of malignancy. These epidemiological and clinical studies clearly establish a link between bile acids in the refluxate and esophageal malignancies. However, the precise molecular mechanisms remain unexplored.

The transcription factor AP-1 is activated by a variety of stimuli and can have both anti-apoptotic and pro-apoptotic functions depending on the cellular context [20]. A correlation between AP-1 and tumorigenesis has been suggested. AP-1 shows increased activity in transformed cell lines [21] and its transactivation is required for tumor promotion *in vivo *[22]. The AP-1 complex is composed of dimers between the Fos (c-Fos, FosB, Fra-1 and Fra-2) and the Jun (c-Jun, JunB, and JunD) family members. Fos and Jun proteins can form heterodimers while only the members of the Jun family are capable of homodimerisation. Fos/Jun heterodimers are more stable than Jun homodimers [23]. AP-1 dimer composition is critical in determining its functional activity and consequently in the induction of specific target genes [20,24,25]. Upstream signalling pathways, mainly mitogen-activated protein kinases(MAPKs), regulate the transcriptional activity and half-life of proteins of the Fos and Jun families giving rise to AP-1 dimers of different transcriptional specificity. Alterations in MAPK signaling have been correlated with malignant progression in humans [26,27]. The MAPK family includes three subfamilies: Erk1/2, p38 and JNK, all of which have been shown to be activated in response to DCA in several cell types including colonic cells, hepatocytes and cholangiocarcinoma cells [11,28].

Cyclooxygenase-2 (COX-2), the rate-limiting enzyme in aracidonic acid metabolism, has been correlated with resistance to apoptosis, inflammation and cancer in several cell types [13,19,29]. COX-2 is upregulated in Barrett's esophagus, esophageal cancer and in animal models of reflux [19,30,31]. COX2 expression can be regulated by MAPKs post-transcriptionally through mRNA stabilization or via activation of AP-1 complexes [32]. Recently, Song *et al. *[33] have demonstrated that unconjugated bile acids such as deoxycholate induced CREB- and AP-1-dependent COX-2 expression in esophageal adenocarcinoma cells and *in vivo *rat model of bile reflux through ROS-mediated activation of PI3K/AKT and ERK1/2. In addition, CREB-specific siRNA and dominant-negative AP-1 (TAM67) blocked deoxycholate- and chenodeoxycholate – induced COX-2 induction. In the present study, we investigated the molecular mechanisms underlying DCA stimulated COX-2 signaling pathway in esophageal adenocarcinoma cells and their possible contribution to deregulated cell survival and apoptosis.

## Methods

### Chemicals

Phorbol 12-myristrate 13-acetate (PMA), acetylsalicidic acid, sodium deoxycholate (DCA) and ursodeoxycholate (UDCA) were from Sigma Chemical Co. (St. Louis, MO). PD 98059 (2'-amino-3'-methoxyflavone), SB 203580 (4-[4'-fluorophenyl]-2 [4'-methylsulfinylphenyl]-5-[4'-pyridyl] imidazole), Z-VAD-FMK (Z-Val-Ala-Asp-CH_2_F), Z-DEVD-FMK (Z-Asp(OCH_3_)-Glu(OCH_3_)-Val-Asp(OCH_3_)-FMK), U0126 (1,4-diamino-2,3-dicyano-1,4-bis [2-aminophenylthio] butadiene), phorbol 12,13-dibutyrate (PdBu) and anisomycin were from Calbiochem (LA Jolla, CA). Poly(dI-dC) and T4 polynucleotide kinase were from Amersham Biosciences (Buckinghamshire, UK).

### Cell culture

The SKGT4 cell line, derived from a well-differentiated adenocarcinoma arising in Barrett's epithelium of the distal esophagus [34] was generously provided by Dr. David Schrump (Bethesda, MA). The gastric adenocarcinoma cell line AGS was from ECACC (Salisbury, UK). Both cell lines were maintained in RPMI 1640 medium supplemented with 10% fetal bovine serum, 4 mM L-Glutamine, 50 units/ml penicillin and 50 μg/ml streptomycin (GIBCO BRL, Life Technologies, Paisley, Scotland) at 37°C in a humidified atmosphere containing 5% CO_2_.

### Electrophoretic mobility shift assay (EMSA)

Control and treated cells were harvested in ice cold phosphate buffered saline (PBS) and nuclear extracts were prepared as described previously [35]. EMSA was performed on nuclear extracts with a double stranded 19-mer oligonucleotides containing the AP-1 binding motif, TGACTCA (12-O-tetradecanoylphorbol-13-acetate response element) as previously described [36]. For supershift analysis, 450 ng of rabbit polyclonal antibodies against c-Jun, Fra-1, and c-Fos or unlabelled oligonucleotides, as a control, were mixed with 4 μg of nuclear extract 30 minutes prior to the binding reaction. Samples were subjected to 4% native polyacrylamide gels. Gels were dried and resulting AP-1 DNA binding complexes visualised by autoradiography.

### Affinity precipitation with biotinylated oligonucleotides

Affinity precipitation of DNA binding proteins was performed with the optimal binding sequence for AP-1 (5'-CGC TTG ATG AGT CAG CCG GAA-3') (Sigma genosys UK) as previously described [37] with the following modifications: total protein content was standardized to 300 – 400 **μ**g/sample using a protein assay (Bio-Rad), according to the manufacturer's instructions. Equal protein content during affinity precipitation was assessed on acetone-precipitated supernatants.

### Total cell lysates

SKGT4 cells were stimulated and total cell lysates obtained using 25 mM Tris-HCl, pH 7.9, 0.2% NP-40, 15 mM NaCl, 1 mM sodium fluoride, 5% glycerol (v/v), 0.05 mM EDTA, 1 mM Na_3_VO_4 _and 1 mM PMSF and 10 μg leupeptin (Sigma) and incubating on ice for 20 minutes. Cell nuclei and debris were eliminated by centrifugation at 10,000 × g for 10 min. The total protein content per sample was standardized to 50–100 μg as above.

### Western blot analysis

Equal amounts of proteins were separated on a 10% SDS-polyacrylamide gel and transferred onto a PVDF membrane (Millipore Corp., Bedford, MA). Membranes were incubated with specific antibodies against Erk1/2, p38 and JNK (at a dilution of 1:1000) (Santa Cruz, CA) or their corresponding phosphorylated forms Erk1/2 (p-Tyr204-Erk1/2), JNK (p-Thr-183/Tyr-185-JNK) (Santa Cruz, CA) and p38 (p-Thr180/Tyr182-p38) (at a dilution of 1:50) (New England Biolabs, Hertfordshire, UK) overnight at 4°C. Antibodies against COX-2 (Cayman chemicals, Alexis Corp, UK) and PARP (Biosource International UK) (at a dilution of 1:100) were also used and incubated overnight at 4°C. Membranes were then incubated with corresponding secondary horseradish peroxidase-conjugated antibodies (Dako, Bucks, UK) (at a dilution of 1:2000) for 1 h at room temperature. Specific immunocomplexes were visualised using the ECL detection system (Amersham Biosciences, Buckinghamshire, UK). For sequential detection, membranes were stripped in 100 mM 2-Mercaptoethanol, 2% SDS, and 62.5 mM Tris pH 6.8 for 30–45 min at 50°C.

### Cell Proliferation assay

SKGT4 cells (2.5 × 10^4^) were plated in a flat-bottomed micro-titre plate and incubated for 24 hr at 37°C and 5% CO_2_. Cells were incubated either with increasing concentrations of DCA (0 – 500 μM) or over a period of 1 – 24 hr with 300 μM DCA. Following stimulation, MTT (3-[4,5-dimethylthiazol-2-yl]-2,5-diphenyl tetrazolium bromide) (Promega Inc., Madison, WI.) was added and cells were further incubated for 1 to 3 hr. Absorbance was measured at 490 nm. Viability is expressed as the percentage of cells remaining in cultures treated with bile acids relative to untreated controls.

### DNA fragmentation ELISA

DCA-induced toxicity was quantified using the cell death detection kit (Roche diagnostics, Penzberg, Germany) according to the manufacturer's standard protocols. Absorbance was measured at 405 nm using an ELISA plate reader.

## Results

### DCA induces AP-1 DNA binding activity in oesophageal cells

**Figure 1:**
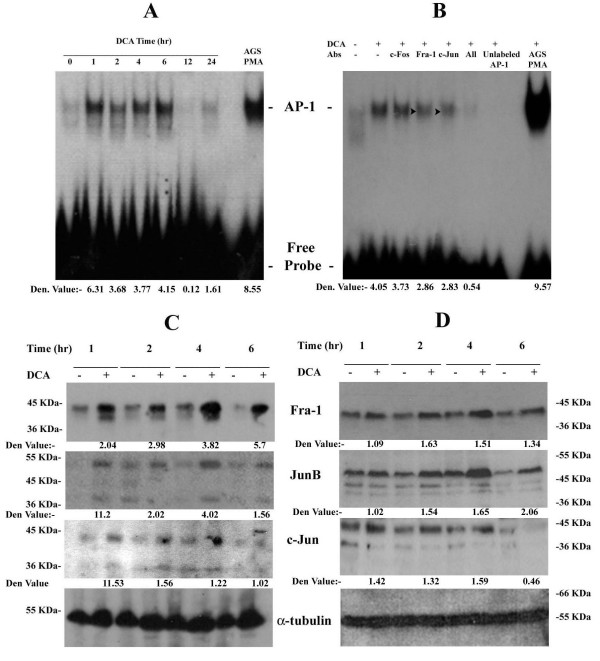
**Fra-1 and c-Jun are members of DCA induced AP-1 Complex**. SKGT4 cells were stimulated with 300 μM DCA for 6 hr (B) or the indicated times (A, C, D). Nuclear extracts (4 μg) were prepared and analyzed for AP-1 binding activity by EMSA (A). For supershift assays the nuclear extracts were incubated for 30 min with 450 ng of antibodies against the various Jun and Fos proteins or with unlabelled probe prior to incubation with the radiolabelled probe. Gastric adenocarcinoma cells (AGS) treated with 50 ng/ml PMA were used as a positive control (B). Total cell lysates were prepared and standardized to 350 μg and DNA affinity purification performed. Affinity purified proteins were resolved by SDS-PAGE and immunoblotted with anti-Fra-1, anti-JunB or anti-c-Jun antibodies, respectively (C). Unbound proteins were immunoblotted with anti-α-tubulin as loading control. Whole cell lysates were standardized to 50 μg and examined by Western blotting as in C (D). Results are representative of at least three independent experiments.

DCA regulates gene transcription through AP-1 activation in colonic cells [28]. We examined the possible link between DCA, AP-1 in esophageal adenocarcinoma SKGT4 cells, a cell line derived from a well-differentiated adenocarcinoma arising in Barrett's epithelium of the distal esophagus [34]. DCA is present at micromolar concentrations (0–300 μM) in esophageal aspirates [18], doses which have been previously shown to be optimal for DCA signaling. SKGT4 cells were exposed to 300 μM DCA from 1 – 24 hr and then analyzed for AP-1 DNA binding activity by EMSA. PMA treated AGS cells were used as a positive control. DCA induces increased AP-1 DNA binding activity as compared to unstimulated cells, in a time dependent manner (Figure 1A). DCA-induced AP-1 activation is biphasic, being markedly induced after 1 hr of stimulation, peaking again at 6 hr and returning to basal levels at the later time points, 12 hr and 24 hr (Figure 1A). We have also demonstrated a similar profile of AP-1 activation in another esophageal adenocarcinoma line OE-33 (data not shown).

### JunB and Fra-1 are the predominant components of DCA-induced AP-1 complex

AP-1 dimer composition is crucial in determining the induction of specific target genes and consequent cellular responses. EMSA was used to determine which Jun and Fos proteins form part of the DCA-induced AP-1 complex. Nuclear extracts from SKGT4 cells stimulated with 300 μM DCA, were incubated with antibodies against c-Fos, Fra-1 and c-Jun prior to incubation with the radiolabelled AP-1 probe. Blocking antibodies that prevent the binding of the corresponding transcription factor rather than causing a supershift were used [38]. The specificity of the DCA-induced AP-1 complex was further verified by the addition of unlabeled AP-1 oligonucleotide. DCA induces clear AP-1 DNA binding activity, which is reduced by addition of antibodies against Fra-1 and c-Jun but not by the anti-c-Fos antibody (Figure 1B). Addition of a pool of all the antibodies completely abrogated the formation of the DCA-induced AP-1 complex. These data suggest that Fra-1 and c-Jun, but not c-Fos, are members of the DCA-induced AP-1 complex.

To further characterize the composition of the DCA-induced AP-1 complex, total cell lysates were prepared from SKGT4 cells treated with 300 μM DCA for 1 – 6 hr and used for DNA affinity precipitation assays with the AP-1 consensus sequence (5'-CGC TTG ATG AGT CAG CCG GAA-3'). Only active forms of the Jun and Fos proteins are able to bind to this oligonucleotide and can be therefore affinity purified and detected by Western blot analysis with specific antibodies. For these assays, we concentrated on c-Jun and Fra-1 as suggested by EMSA and also included JunB and JunD, as they have been shown to respectively counteract or enhance c-Jun activity [21]. These experiments show that DCA stimulates a time dependent increase in Fra-1, JunB and c-Jun DNA binding activity (Figure 1C). No activation of JunD was observed at any stimulation time (data not shown). DCA induces strong Fra-1 binding activity after 1 hr, which is sustained for at least 6 hr of stimulation. Fra-1 is detected as two bands with distinct electrophoretic mobility: a slower migrating, more prominent band and a fainter faster migrating band (Figure 1C). After prolonged stimulation, the slower species is stabilized while the faster migrating species is no longer detected (Figure 1C). These two electrophoretic mobility forms of Fra-1, which most likely correspond to different phosphorylation states, have been previously reported in other cell types [39]. Similarly, DCA-induced JunB activity is clear at 1 hr and remains elevated for up to 6 hr of stimulation (Figure 1C). On the other hand, DCA induces a weak and more transient activation of c-Jun, which is maximal at 4 hr and is consistently weak and even negligible at 6 hr (Figure 1C). These data indicate that DCA induces AP-1 complexes composed of Fra-1, JunB and c-Jun at early stages of stimulation, but only of Fra-1 and JunB at 6 hrs. Fos and Jun proteins can form heterodimers while the only the members of the Jun family can homodimerise [23]. Therefore, the possible types of early induced complexes are c-Jun/c-Jun, c-Jun/JunB, c-Jun/Fra-1, JunB/Fra-1 or JunB/JunB, while only the latter two would be present at later stages.

### DCA enhances the basal expression levels of Fra-1, JunB and c-Jun proteins

Induction of AP-1 DNA binding activity can be achieved by activation of pre-existing Fos/Jun proteins or through induction of *de novo *protein expression [40]. To differentiate between these two possibilities, the protein expression levels of these molecules was assessed by Western blotting in SKGT4 cells following DCA treatment (300 μM) for 1 – 6 hr. SKGT4 cells express basal levels of Fra-1, JunB and c-Jun (Figure 1D). The expression levels of all three proteins are further enhanced by DCA treatment. An increase in Fra-1 and JunB protein levels is observed within 1 hour of stimulation and remains constant for up to 6 hours (Figure 1D). DCA induces a lesser increase in c-Jun protein expression as compared to Fra-1 and JunB, which decreases by 6 hours (Figure 1D). JunB is detected as three distinct bands while c-Jun is generally found as a doublet. Multiple electrophoretic mobility forms of JunB and c-Jun attributed to different phosphorylation status have previously been reported [40]. The presence of basal expression levels together with the matching kinetics of enhanced protein expression and those of DNA binding activity for Fra-1, JunB and c-Jun, suggest that DCA induces AP-1 DNA binding activity through activation of pre-existing molecules as well as either induction of *de novo *protein synthesis or increased protein stability. Sustained activation of AP-1 components has been associated with oncogenic transformation [41]. As c-Jun is only transiently activated by DCA, we concentrated on Fra-1 and JunB in subsequent experiments.

### AP-1 is induced by DCA at concentrations found in Barrett's esophagus

**Figure 2:**
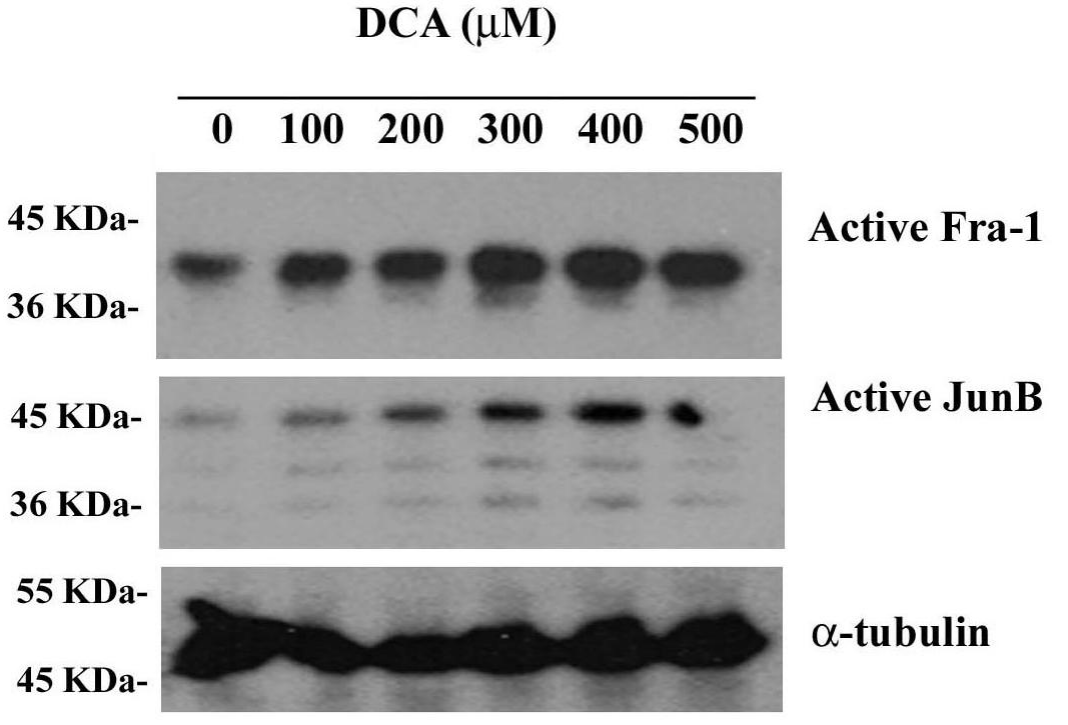
**AP-1 is induced by DCA at concentrations found in Barrett's oesophagus**. SKGT4 cells were treated with DCA over a concentration range of 0 – 500 μM for 6 hr. Total cell lysates were prepared and standardized to 350 μg. DNA affinity purification was performed and proteins were resolved by SDS-PAGE and immunoblotted with anti-Fra-1 or anti-JunB, respectively. Unbound proteins were immunoblotted with anti-α-tubulin as loading control. Results are representative of at least three independent experiments.

Increased concentrations of bile acids (> 200 μM) associated with higher severity of disease, have been observed in esophageal aspirates in patients with erosive esophagitis and Barrett's esophagus [16,18]. The contribution of various doses of DCA (0 – 500 μM) following prolonged stimulation (6 hours) was examined on Fra-1 and JunB DNA binding activity in SKGT4 cells using the affinity precipitation assay. DCA induces a dose dependent increase in the DNA binding activity of Fra-1 and JunB at 6 hours of stimulation (Figure 2). Low concentrations of DCA stimulate a modest increase while stronger activation of Fra-1 and JunB is detected at and above 300 μM DCA (Figure 2). These data show that strong activation of AP-1 is achieved by DCA at the concentrations observed *in vivo *in patients with Barrett's esophagus [18].

### DCA induces sustained activation of Erk1/2 and p38 but not of JNK

**Figure 3:**
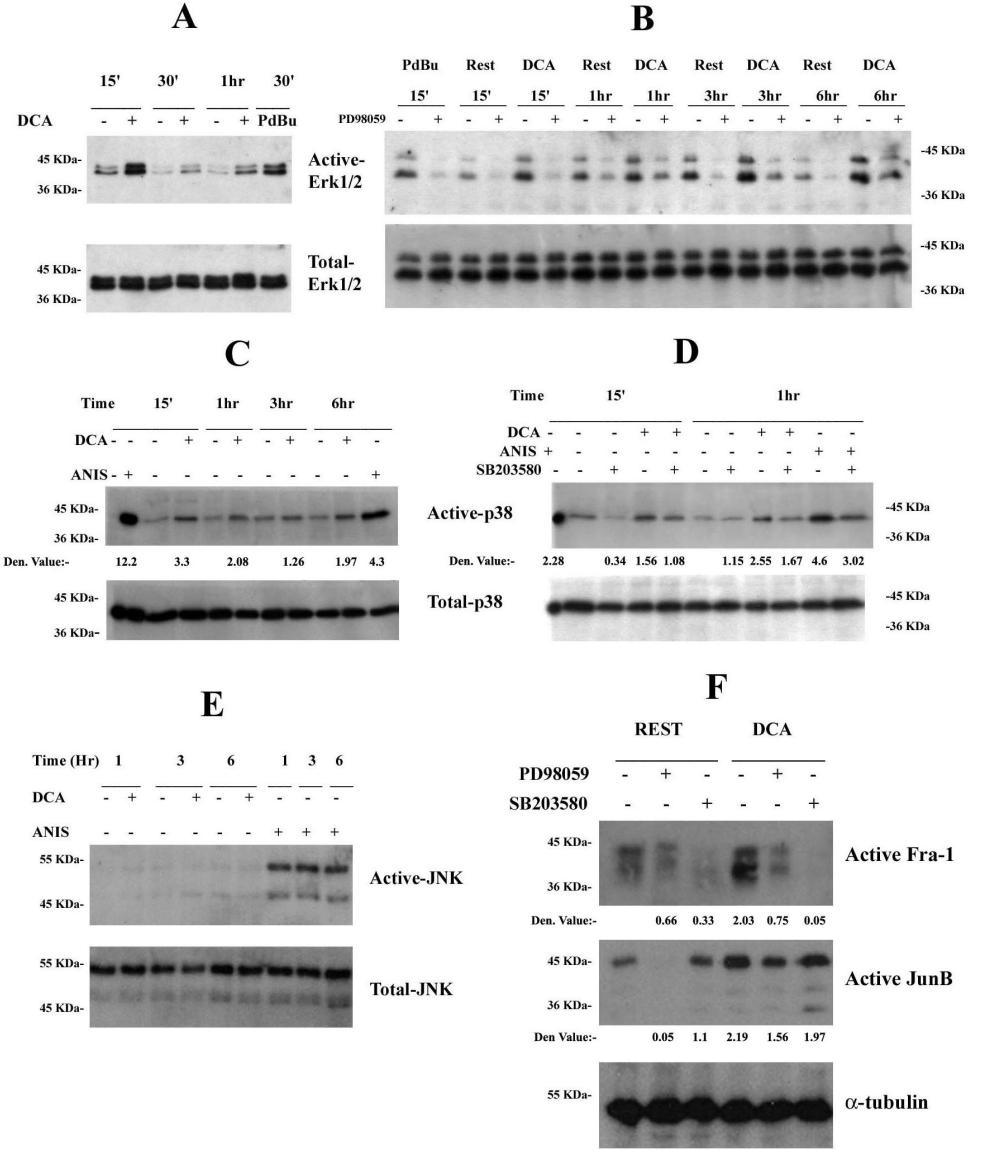
**DCA induces sustained activation of Erk1/2 and p38 but not of JNK**. SKGT-4 cells were treated with 300 μM DCA for indicated times. Treatment with 50 ng/ml PdBu for 30 min (A, B) or 10 μg/ml anisomycin (ANIS) for 6 hr (C, D, E) were used as positive controls for Erk or p38 and JNK activation, respectively. In panel C the first two lanes correspond to unstimulated and anisomycin stimulated C-6 glioma cells (positive control). Where indicated, cells were incubated in the presence or absence of 50 μM PD98059 (B, F) or 2 μM SB203580 (D, F) for 30 min prior to stimulation. Total cellular proteins were standardized to 50 μg and assessed by Western blotting using antibodies specific for phospho-Erk1/2 and total Erk1/2 (A, B); phospho-p38 and total p38 (C, D); or phospho-JNK and total JNK (E). Total cell lysates were prepared and standardized to 350 μg. DNA affinity purified proteins were resolved by SDS-PAGE and immunoblotted with anti-Fra-1 or anti-JunB, respectively (F). Unbound proteins were immunoblotted with anti-α-tubulin as loading control. Results are representative of at least three independent experiments.

AP-1 activation is mainly regulated by MAPKs. We therefore examined the ability of DCA to activate Erk1/2, p38 and JNK in SKGT4 cells using Western blot analysis with specific antibodies that recognize the active phosphorylated forms of these proteins: Erk1/2 (p-Tyr204-Erk1/2), p38 (p-Thr180/Tyr182-p38) and JNK (p-Thr-183/Tyr-185-JNK). The well known Erk1/2, p38 and JNK activators phorbol 12, 13-dibutyrate (PdBu) and anisomycin (ANIS) were respectively used as positive controls (Figure 3A–E). Time course analyses show that 300 μM DCA induces sustained activation of Erk1/2 and p38, which are detected as early as 15 minutes and persist for at least 6 hr of stimulation (Figures 3A–C), while JNK is not activated at any time tested (Figure 3E). DCA does not influence the protein expression levels of any of these MAPKs (Figure 3A–E lower panels).

Mek1/2 and MKK3/6 are the respective upstream activators of Erk1/2 and p38 [42,43]. The PD98059 and SB203580 compounds, known specific inhibitors of Mek1/2 and MKK3/6 were used to verify activation of Erk1/2 and p38 in response to DCA. SKGT4 cells were incubated with 50 μM PD98059 or 2 μM of the SB203580 for 30 minutes prior to the addition of 300 μM DCA. PD98059 completely abolishes basal, PdBu and DCA-induced Erk1/2 activity at all the time points tested (Figure 3B). Similarly, the SB203580 compound inhibits the activation of p38 in response to DCA and to anisomycin at all tested time points (Figure 3D). These data show that DCA activates the MAPKs Erk1/2 and p38 without affecting their protein expression levels, but it is unable to regulate JNK activation or protein expression.

### DCA mediates AP-1 DNA binding through activation of Erk1/2 and p38

The pharmacological inhibitors PD98059 and SB203580 were respectively used to corroborate the contribution of the Raf-Mek1/2-Erk1/2 and the MKK3/6-p38 pathways in DCA-induced DNA binding of Fra-1 and JunB. SKGT4 cells were pre-treated with 10 μM PD98059 or 2 μM SB203580 for 30 min prior to stimulation with 300 μM DCA for 6 hr and DNA affinity precipitation assays were performed. Pre-treatment of SKGT4 cells with 10 μM PD98059 impairs and diminishes DCA-induced activation of Fra-1 and JunB, respectively (Figure 3F). The SB203580 compound completely abolishes DCA-induced Fra-1 DNA binding while having no effect on DCA-induced JunB DNA binding (Figure 3F). These data indicate that both Raf-Mek1/2-Erk1/2 and MKK3/6-p38 are involved in DCA-induced Fra-1 activation, while only Raf-Mek1/2-Erk1/2 is upstream of JunB activation.

### DCA induces a decrease in cell proliferation that is accompanied by low levels of apoptosis

**Figure 4:**
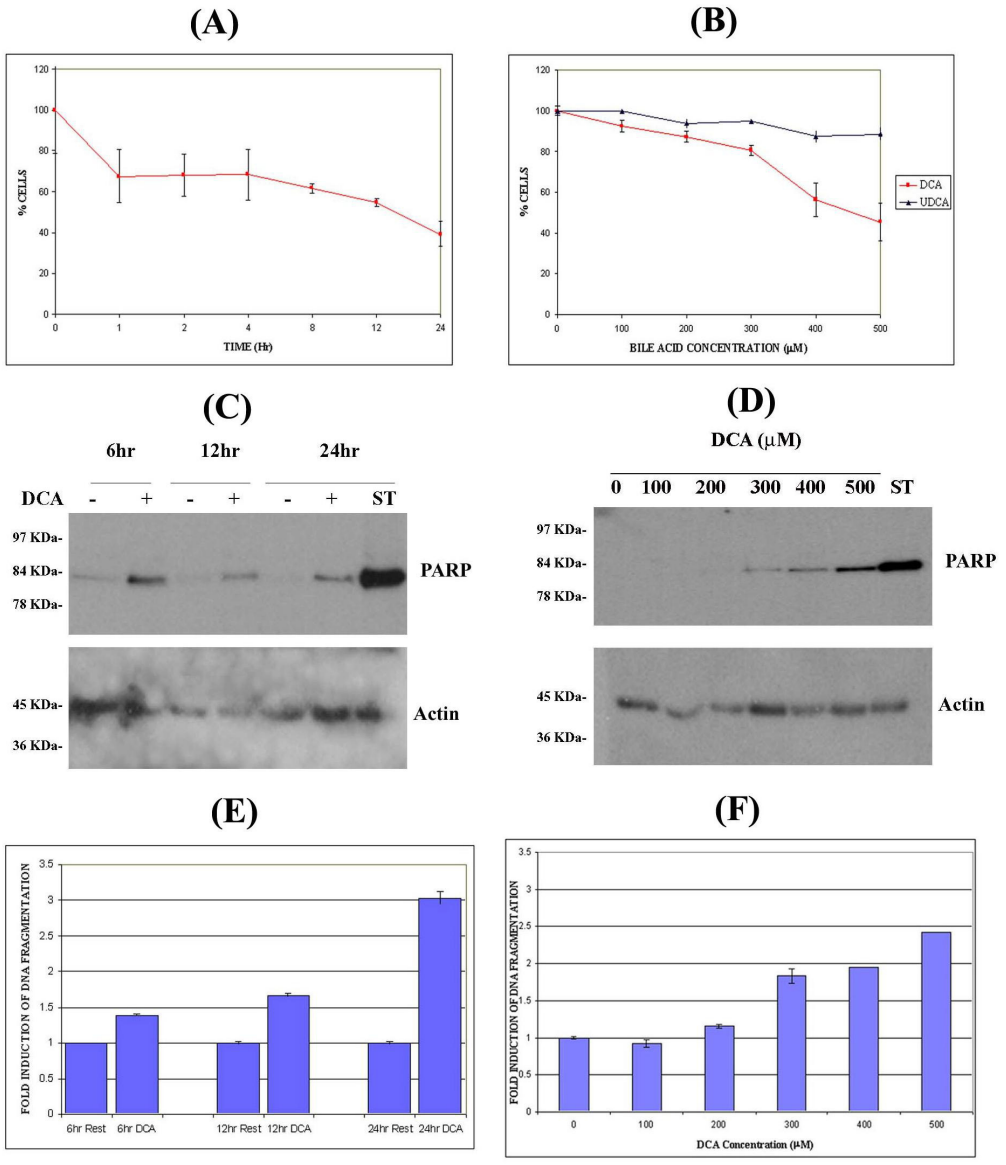
**DCA induces PARP cleavage and DNA fragmentation with reduced cell proliferation in SKGT4 cells**. SKGT4 cells were incubated with 300 μM DCA at the indicated time intervals (A) and at DCA concentrations ranging from 0 – 500 μM for 6 hours (B). In panels A and B, cellular proliferation was assessed using the MTT assay as described in experimental procedures. Results are expressed as percentage of proliferating cells relative to untreated controls. Mean ± SD. To investigate DCA induced PARP cleavage (C,), SKGT4 cells were stimulated with 300 μM DCA for the indicated times or with 0 – 500 μM DCA for 6 hr (D). 1 μM Staurosporine (ST) was used as positive control. In panels C and D, total cell lysates were standardized to 50 μg as described in experimental procedures and assessed by Western blotting using an antibody directed against cleaved PARP to detect apoptosis. Anti-actin antibody used as loading control. SKGT4 cell lysates were assessed for DNA fragmentation as described in Methods (E, F). Results are presented as fold induction of DNA fragmentation relative to unstimulated control. Mean ± SD.

Bile acids, in particular DCA, inhibit proliferation and are potent inducers of apoptosis in several cell types including, hepatocytes and colonic cells [10,13,28]. Activation of AP-1 can have both anti-apoptotic and pro-apoptotic functions depending on the cellular context [20]. Since DCA induces sustained (6 hour) activation of AP-1 in SKGT4 cells (Figure 1A), its possible contribution to deregulated cell survival and apoptosis was examined. SKGT4 cells were stimulated with 300 μM DCA or 300 μM ursodeoxycholic acid (UDCA) for 0 – 6 hr. We have previously shown that UDCA, in contrast to DCA, does not induce AP-1 transcription factor activation in colon cancer cells. In fact, it inhibits interleukin-1 beta and deoxycholic acid-induced activation of NF-kappaB and AP-1 in these cells [44]. Cell proliferation was assessed using the MTT assay. DCA induces a dose and time dependent decrease in cellular proliferation, which is initially observed within the first hour of treatment, remains at similar levels up to 8 hr and is more pronounced at 12 and 24 hr (Figure 4A). This decrease is clear at 300 μM DCA and higher concentrations, being statistically significant (p < 0.05) at 400 – 500 μM (Figure 4B). Dramatic morphological changes indicative of apoptosis are also observed at 6 hr of DCA treatment at concentrations in excess of 300 μM (data not shown). In comparison, cells stimulated with UDCA show identical proliferation patterns and morphology as compared to untreated cells at all times and concentrations tested.

DNA fragmentation and PARP cleavage, two of the hallmarks of apoptosis, were respectively assessed by quantifying cytoplasmic histone-associated DNA fragments (mono and oligonucleosomes) by ELISA and Western blotting using a specific antibody that recognizes the 85 kDa cleaved PARP fragment. DCA dose response and kinetic studies showed that a low level of PARP cleavage was detected at 6 hr post DCA treatment and persisted for up to 24 hr (Figure 4C). This effect is dose-dependent, being observed at 300 – 500 μM DCA but not at lower concentrations (Figure 4D). Similarly, DCA induced a 1.5-fold increase in DNA fragmentation after 6 hr, which increased after 24 hr (3-fold) (Figure 4E). DNA fragmentation with low concentrations of DCA (100 – 200 μM) was similar to resting cells, while increasing concentrations (300 – 500 μM) resulted in a steady rise in DNA damage (Figure 4F). Taken together, these data show that DCA induces a reduction in cell proliferation, which is accompanied by low levels of apoptosis. These effects are sustained and dose dependent, being observed at high DCA concentrations similar to those found in patients with erosive esophagitis and Barrett's esophagus [16,18].

### DCA-induced PARP cleavage is caspase-dependent

**Figure 5:**
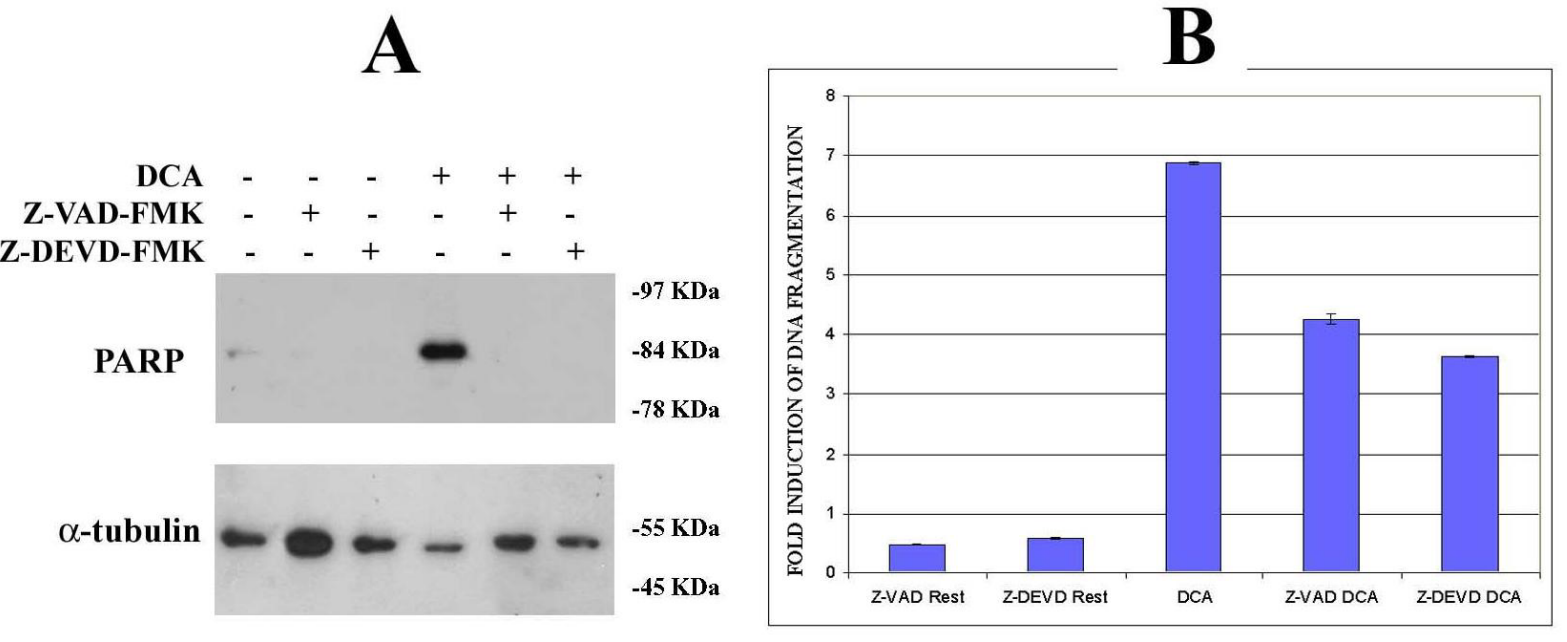
**DCA-Induced PARP cleavage and DNA fragmentation is Caspase Dependent**. SKGT4 cells were treated with 50 μM Z-VAD-FMK or 50 μM Z-DEVD-FMK for 1 hr prior to the addition of 400 μM DCA for 6 hr. Whole cell lysates were standardized to 50 μg as described in experimental procedures. PARP cleavage was assessed by immunoblotting with an anti-PARP antibody followed by anti-α-tubulin as loading control (A). DNA fragmentation was assessed by ELISA (B). Results are given as fold induction of DNA fragmentation relative to unstimulated cells. Mean ± SD. Results are representative of at least two independent experiments.

PARP cleavage can occur via caspase-dependent and independent mechanisms [45]. The broad-spectrum caspase inhibitor, Z-Val-Ala-Asp-CH_2_F (Z-VAD-FMK), and the specific caspase-3 inhibitor, Z-Asp(OCH_3_)-Glu(OCH_3_)-Val-Asp(OCH_3_)-FMK (Z-DEVD-FMK), were employed to assess the role of caspases in DCA-induced PARP cleavage. SKGT4 cells were pretreated for 1 hr with 50 μM of either Z-VAD-FMK or Z-DEVD-FMK and stimulated with 400 μM DCA, a concentration which induced significant levels of PARP cleavage (Figure 4) for 6 hr. Unstimulated SKGT4 cells showed negligible levels of PARP cleavage and DNA fragmentation. Both Z-VAD-FMK and Z-DEVD-FMK completely abolished DCA-induced PARP cleavage while partially inhibiting DNA fragmentation (Figures 5A and 5B). These data indicate that DCA-induced PARP cleavage is caspase-3 dependent, while DNA fragmentation is only partially dependent on this pathway.

### DCA-induces COX-2 expression via Erk1/2 and p38-dependent mechanisms

**Figure 6:**
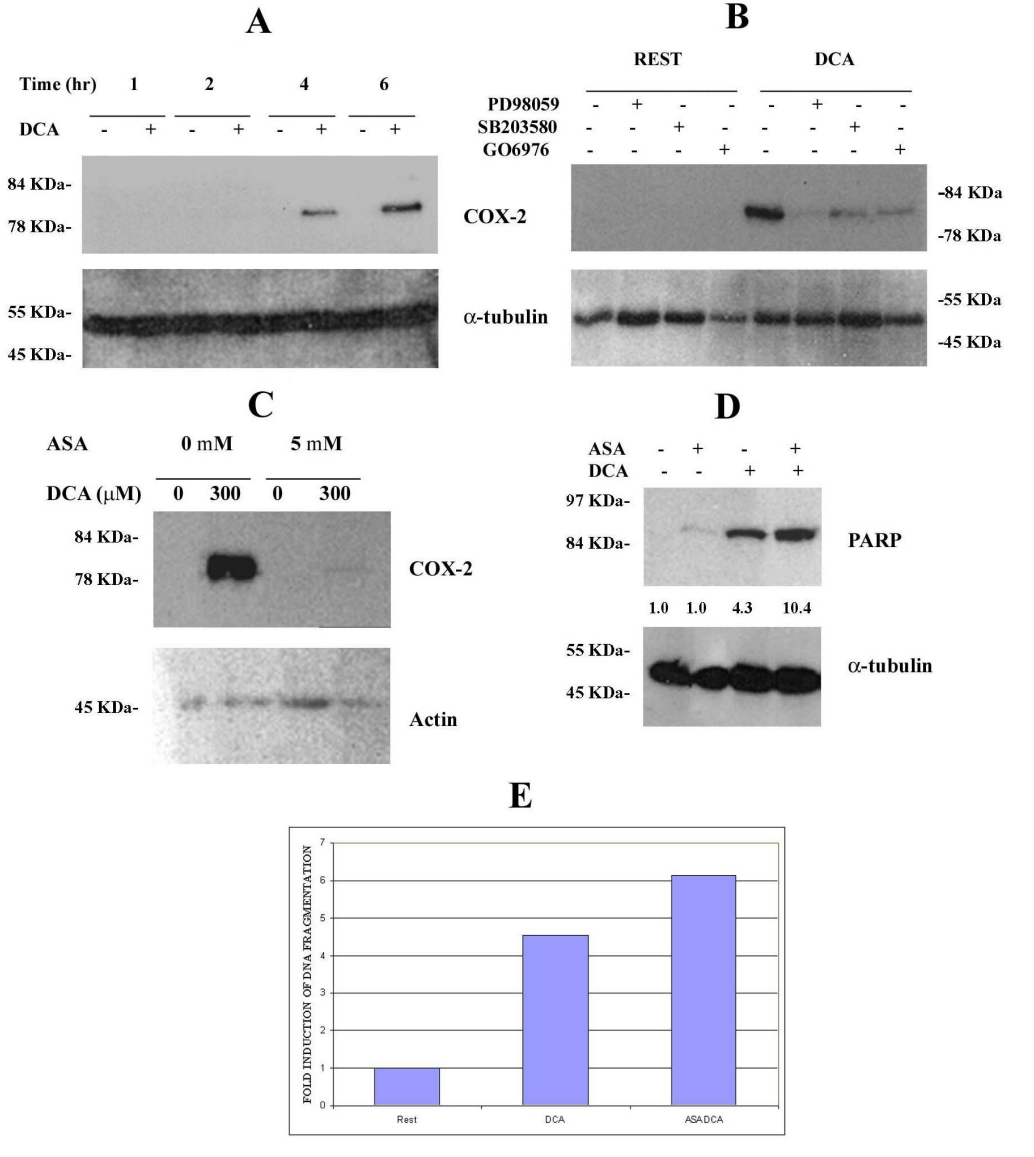
**DCA regulates SKGT4 cell survival through induction of COX-2 expression via Erk1/2 and p38-dependent pathways**. SKGT4 cells were stimulated with 300 μM DCA for 1–6 hr and analyzed for COX-2 expression by Western blot analysis (A). SKGT4 cells were treated with 10 μM PD98059, 2 μM SB203580 or 1 μM GÖ6976 for 30 min prior to the addition of 300 μM DCA for 6 hr followed by analysis of COX-2 induction (B). SKGT4 cells were treated with 5 mM acetylsalicylic acid (ASA) for 30 min preceding stimulation with 300 μM (C) or 400 μM (D) DCA for 6 hr. In panels A-D, total cell lysates were assessed by Western blotting using either anti-COX-2 or anti-PARP antibodies. anti-actin or anti-α-tubulin antibody used as loading control. In panels C and D, COX-2 expression and PARP cleavage were assessed by densitometry and normalised against actin or α-tubulin, respectively. Fold increases in COX-2 expression and PARP cleavage are given relative to resting cells. ELISA was utilized to assess DNA fragmentation (E). Results are given as fold induction of DNA fragmentation relative to unstimulated cells. Results are representative of three independent experiments.

Interestingly, the levels of DCA-induced PARP-cleavage plateau and do not increase progressively. This suggests that a compensatory survival mechanism might be concomitantly regulated by DCA. Enhanced protein expression of COX-2 has been correlated with cellular proliferation and resistance to apoptosis in various cell types [13,19]. Therefore the induction of COX-2 protein expression by DCA in SKGT4 cells was examined using Western blot analysis. COX-2 is not expressed in unstimulated cells, but it is readily induced after 4 hr of DCA stimulation (Figure 6A). Maximal induction is achieved at 6 hours with 300 μM DCA (Figure 6A). In agreement with previous reports, COX-1 protein is not constitutively expressed in this cell line.

COX-2 protein expression can be regulated at transcriptional and posttranscriptional levels by MAPKs and by AP-1 through binding to the CREB site in the COX-2 gene promoter in various cell types [29,32]. Since DCA induces AP-1 activity through the activation of Erk1/2 and p38 (Figure 6B), we explored the involvement of these pathways in the regulation of COX-2 protein expression in our system. SKGT4 cells were pre-treated with 10 μM PD98059, 2 μM SB203580 or 1 μM Go6976 for 30 minutes prior to addition of 300 μM DCA for 6 hr. (GO6976 was used as a positive control in this experiment as it has previously been shown to inhibit COX-2 expression [46]. The induction of COX-2 protein expression in response to DCA was strongly inhibited by all three compounds (Figure 6B). These results demonstrate that the Erk1/2 and p38 pathways are not only responsible for DCA-induced Fra-1 and JunB activation but also for induction of COX-2 protein expression.

### DCA-induced COX2 regulates apoptotic markers in SKGT4 cells

The kinetics of COX-2 protein induction in response to DCA correlates with those of PARP cleavage and DNA fragmentation (Figures 4 and 6A). To examine the possible anti-apoptotic role of COX-2 in our system, the induction of COX-2 expression was inhibited using acetylsalicylic acid (ASA) and the levels of apoptosis assessed. Treatment of SKGT4 cells with 5 mM ASA for 30 min prior to the addition of 400 μM DCA resulted in a dramatic reduction in COX-2 expression in response to DCA (Figure 6C). DCA induced clear PARP cleavage in comparison to untreated cells, an effect that was enhanced by pre-treatment with ASA (Figure 6D). Levels of DNA fragmentation were similar in unstimulated cells and cells treated with ASA alone. DCA induced a four-fold increase in DNA fragmentation that was further increased by pre-treatment with ASA (Figure 6E). These data show that inhibition of COX-2 expression readily enhances the apoptotic markers induced upon DCA exposure, confirming a potential anti-apoptotic role of COX-2 in this system.

## Discussion

Bile acids are major constituents of the gastroesophageal refluxate and are regarded to have an important role in malignant development in the esophagus. [15,17]. A significant proportion of the bile acids in patients with extensive mucosal injury was composed of the dehydroxylated taurodeoxycholic acid and the unconjugated cholic and deoxycholic acids. Increased concentrations of bile acids (> 200 μM) have been observed in esophageal aspirates in patients with erosive esophagitis and Barrett's esophagus. [15,16,18]. The exact molecular mechanism by which bile acids contribute to this process has not been defined. Alterations in gene expression underlie the ability of deoxycholate to deregulate biochemical processes and control the fate of the cells. The use of *in vitro *cell culture model as in our study may be at variance with how cancer behaves in humans. However, the analysis of genes and molecules that are important in cancer development in cancer cell lines is of importance to our understanding of our interpretation of the *in vivo *situation.

The purpose of this study was to investigate the mechanisms by which deoxycholate stimulates COX-2 and AP-1 expression and the role of COX-2 in the mediation of pro-apoptotic and anti-apoptotic mechanisms. Using electrophoretic mobility shift assays, we demonstrated that DCA induced persistent AP-1 DNA binding activity. AP-1 activation results from dimerisation of either pre-existing or newly synthesised phosphorylated proteins of the Fos and Jun families. Fra-1 and JunB are the predominant components of the sustained AP-1 complex, while c-Jun is only transiently induced. Interestingly, this AP-1 dimer composition is distinct from that induced by DCA in colonic epithelial cells where the induced complex contains JunD, Fra-1 and c-Fos [47]. DCA-induced activation of Fra-1 was dependent on both the Mek1/2-Erk1/2 and p38 pathways, while JunB activation was mediated solely through the Mek1/2-Erk1/2 cascade in esophageal cells. It has previously been demonstrated that levels of Erk1/2 activity are greater in Barrett's esophagus than in GERD [48]. In addition, duration of Erk1/2 activation determines composition and transcriptional output of AP-1 [49]. Our data are in agreement with previous reports showing that sustained activation of Erk1/2 results in Fra-1 and JunB activation with negligible induction of c-Jun [39].

The precise mechanisms utilized by duodenal reflux to elicit esophageal damage and promote tumorigenesis are uncertain. Accumulating evidence suggests that COX-2 is involved in the development of Barrett's esophagus and esophageal adenocarcinoma. COX-2 is frequently overexpressed in esophageal adenocarcinoma cells and tissues. [19,30,31]. Song *et al. *[33] reported that the unconjugated bile acids chenodoxycholate and deoxycholate potently upregulate ROS production in the esophagus, leading to activation of the PI3K and ERK1/2 signaling pathways, with a subsequent CREB- and AP-1-dependent COX-2 expression. Here, we demonstrate a significant role for COX-2 in mediating survival in these cells, which is dose and time dependent. Exposure to DCA results in inhibition of proliferation with concomitant induction of low levels of apoptosis. Furthermore, DCA induces a dose- and time-dependent increase in COX-2 expression that parallels with PARP cleavage and DNA fragmentation. DCA-induced apoptosis is both dose- and time-dependent and requires caspase-3 activation. Furthermore the activation of Erk1/2 and p38 is crucial for DCA-induced COX-2 expression, an AP-1 target gene. Our findings strongly suggest that DCA induces pro- and anti-apoptotic signaling cascades and their combined activity determines cell fate.

Previous studies from our laboratory and others have demonstrated that DCA can induce NF-κB in esophageal cells [50,51]. DCA-induced PARP cleavage is dependent on caspase-3 activation [52]. Therefore, simultaneous activation of caspase-3 and NF-κB explains the observed low levels of PARP cleavage induced by DCA. Glinghammar *et al. *[53] observed that in response to DCA, colonic cells undergo apoptosis and have low caspase-3 activation, strong activation of NF-κB and AP-1 transcription factors, and COX-2 expression. In agreement with these findings, we have demonstrated that SKGT4 cells exposure to DCA resulted in low levels of caspase-3-dependent PARP cleavage, activation of NF-κB and AP-1, and substantial induction COX-2 expression.

AP-1 dimer composition is critical in determining its functional activity and consequently in the induction of specific target genes [20,24,25]. The Fos family members and c-Jun are positive regulators of cell proliferation and have been shown to mediate oncogenic transformation in fibroblasts [54]. In the absence of c-Jun in mouse embryonic fibroblasts, JunB acts as a positive growth regulator [20]. However, when both molecules are expressed, JunB prevents c-Jun DNA binding, transactivation and consequent transformation potential [55,56]. Hence as both molecules are constitutively expressed in SKGT4 cells, the enhanced levels of JunB induced by DCA might potentially have a negative effect on c-Jun DNA binding. Fos and Jun proteins can heterodimerise while only the members of the Jun family are capable of homodimerisation. Fos/Jun heterodimers are more stable than Jun homodimers [23]. Therefore, our data suggest that a Fra-1/JunB heterodimer is the DCA-induced AP-1 complex. In these circumstances, this complex could act as a growth promoter in response to DCA in esophageal cells.

Bile acids can exert their tumor promoting activity by affecting intracellular signaling pathways, which alters proliferation and apoptosis. MAPKs constitute an important group of signaling mediators that govern cellular processes such as proliferation and cell death. DCA promotes cell survival through the induction of MAPKs in primary hepatocytes and colonic cells [11,28]. The present study demonstrated that DCA activated the MAPKs Erk1/2 and p38, but it is unable to regulate JNK activation. COX-2 expression can also be regulated by MAPKs both directly by mRNA stabilization as shown in intestinal epithelial cells and monocytes [29,32,57] and indirectly through activation of AP-1 complexes [33]. Here we used specific pharmacological inhibitors of the MAPK cascades to identify the pathways mediating DCA-induced COX-2 expression in SKGT4 cells. COX-2 expression was completely blocked by the PD98059 and SB203580 compounds demonstrating the involvement of Raf-Mek1/2-Erk1/2 and MMK3/6-p38 pathways in DCA-induced COX-2 expression. COX-2 may be specifically important in esophageal carcinogenesis, as COX-2 expression is frequently upregulated in Barrett's esophagus, esophageal cancer and in animal models of reflux [19,30,31]. The specificity of pharmacological inhibitors should be considered, because many of these inhibitors block various signal transduction proteins. However, MAPKs inhibitors such as PD98059 and SB203580 are well-established specific inhibitors of ERK and p38 pathway, respectively, and have been tested in many systems for their specificity in inhibiting MAPKs activity.

*In vitro*, prolonged exposure of colonic cells to DCA induced apoptosis and caused morphological changes that were characteristic of apoptosis [10], however, apoptosis resistant clones may be selected after frequent exposure to the cytotoxic bile DCA [58]. *In vivo*, bile acid-induced apoptosis has been linked with compensatory proliferation of crypt epithelial cells [59]. Rates of apoptosis have been demonstrated to be low in Barrett's epithelium [60], potentially contributing to the malignant process. While DCA induced low levels of apoptosis in this study, the effects on cell survival appear to have been balanced at least partially through DCA-induced COX-2. Specifically DCA-induced PARP cleavage was enhanced by COX inhibition. However, other MAPK-regulated pathways such as induction of the anti-apoptotic proteins Mcl-1 and cFLIP and Bcl-2 might also contribute to esophageal cell survival in response to DCA [61].

Reflux of duodenal contents appears to contribute to the development of esophagitis and Barrett's adenocarcinoma [15,17] DCA-induced sustained AP-1 activation is likely to have important implications in esophageal tumorigenesis considering that blockage of DMBA (7,12-dimethylbenz [*a*]anthracene)/PMA-induced AP-1 activity in transgenic mice has been demonstrated to prevent neoplastic transformation in a murine keratinocyte model [22]. DCA stimulation also results in sustained expression of the anti-apoptotic protein COX-2. Long-term intermittent exposure of esophageal tissue to DCA such as that caused by duodenal reflux will therefore likely lead to sustained MAPK and AP-1 activation, as well as over-expression of COX-2. Persistent activation of MAPK can lead to enhanced cell proliferation possibly via cyclin D1 expression [62]. It is well known that MAPKs regulate the downstream phosphorylation of nuclear transcription factors such as AP-1 and NF-κB, which regulate several cellular events including apoptosis and proliferation. Cytokines that are stimulated by NF-κB, such as IL-1β and TNF-α, released in response to chronic gastroesophageal reflux, can also directly activate the AP-1 and NF-κB pathway.

## Conclusion

In conclusion, the experiments presented here clearly demonstrate that MAPKs and AP-1 participate in the regulation of COX-2 expression. The combination of these events might be responsible for shifting the DCA-regulated apoptosis/survival balance towards the acquisition of an apoptosis resistant phenotype, as that associated with the progression from Barrett's metaplasia to adenocarcinoma [63]. This model is in agreement with previous data showing that sustained activation of AP-1 and COX-2 are associated with increased invasion and oncogenic transformation [22]. The present report strengthens the argument that bile acid reflux is important in malignant progression in Barrett's patients.

## List of abbreviations

ANIS: anisomycin; AP-1: activator protein-1; ASA: acetylsalicylic acid; COX-2: cyclooxygenase-2; DCA: deoxycholic acid; DMBA: 7,12-dimethylbenz [*a*]anthracene; GERD: gastroesophageal reflux disease; Erk: extracellular signal regulated kinase; MAPK: mitogen activated Protein Kinase; PdBu: phorbol 12, 13-dibutyrate; PMA: phorbol 12-myristate 13-acetate.

## Competing interests

The authors declare that they have no competing interests.

## Authors' contributions

EL carried out the study and prepared the manuscript. MAL participated in the design of the study experimentation and edited the manuscript. VAM made contributions to the study methodology of MAPK and AP-1 analysis. SD contributed to the design of the study experimentation and drafting of the manuscript. AL coordinated the study and edited the manuscript. DK designed and supervised the study. All authors have read and approved the final manuscript.

## Pre-publication history

The pre-publication history for this paper can be accessed here:

http://www.biomedcentral.com/1471-2407/9/190/prepub
